# Manufacturing a Micro-model with Integrated Fibre Optic Pressure Sensors

**DOI:** 10.1007/s11242-018-1000-y

**Published:** 2018-01-11

**Authors:** I. M. Zarikos, S. M. Hassanizadeh, L. M. van Oosterhout, W. van Oordt

**Affiliations:** 1 Department of Earth Sciences, Utrecht University, Budapestlaan 4, 3584 CD Utrecht, The Netherlands; 2 Department of Chemical Engineering, Delft University of Technology, van der Maasweg 9, 2629 HZ Delft, The Netherlands

**Keywords:** Two-phase flow, Micro-model, Pore-scale pressure measurement

## Abstract

The measurement of fluid pressure inside pores is a major challenge in experimental studies of two-phase flow in porous media. In this paper, we describe the manufacturing procedure of a micro-model with integrated fibre optic pressure sensors. They have a circular measurement window with a diameter of 260 $\upmu \hbox {m}$, which enables the measurement of pressure at the pore scale. As a porous medium, we used a PDMS micro-model with known physical and surface properties. A given pore geometry was produced following a procedure we had developed earlier. We explain the technology behind fibre optic pressure sensors and the procedure for integrating these sensors into a micro-model and demonstrate their utility for the measurement of pore pressure under transient two-phase flow conditions. Finally, we present and analyse results of single and two-phase flow experiments performed in the micro-model and discuss the link between small-scale fast pressure changes with pore-scale events.

## Introduction

Two-phase flow in porous media is a process encountered in various applications such as oil recovery, (Muskat 1949; Melrose and Brandner 1974), soil remediation (Mayer and Hassanizadeh 2005), $\hbox {CO}_{2}$ sequestration (Uemura et al. 2011; Kazemifar et al. 2015) and many other industrial systems.

Multiphase flow in porous has been experimentally investigated with the use of micro-models, as well as natural porous media. The advantage of micro-models over natural porous media is that the medium characteristics, such as pore size, pore connectivity, wettability, and pore–throat aspect ratio, are fully controlled and defined. Apart from the well-defined properties of the medium, micro-models are advantageous compared to natural porous media, mostly because they allow for direct visualization. Traditional imaging techniques, such as microscopy, offer high data acquisition rate allowing the monitoring of pore-scale dynamic phenomena, which may occur in less than one second. Thus, processes such as disconnection and remobilization of the non-wetting phase can be monitored and better understood. The extended use of micro-models in two-phase flow studies has led to the development of various manufacturing techniques and micro-models from different materials and with different properties (see Karadimitriou and Hassanizadeh 2012, for a review). Typically, micro-models have been made of glass (Wardlaw and Yu 1988; Ioannidis et al. 1991; Sahloul et al. 2002; Karadimitriou et al. 2012), silicone (Zhang et al. 2011; Wang et al. 2013) and polydimethylsiloxane (PDMS) (Karadimitriou et al. 2013, 2014a, b; Zhang et al. 2013; Kunz et al. 2015).

Up to now, fluid pressure measurements during two-phase flow experiments in micro-models have been mainly performed in external lines or in inlet/outlet areas of the micro-model. For example, absolute or differential pressure between the inflow and outflow reservoirs of the micro-model has been measured with dedicated pressure sensors (Karadimitriou et al. 2014a, b; Tsakiroglou et al. 2007; Kunz et al. 2015). However, external pressure measurements, despite being valuable, are not representative of the pressure distribution inside the micro-model pore space.

Pressure measurements at the pore scale are challenging, and the current technology is limited to a few potentially applicable techniques, such as pressure-sensitive paints (PSP) and atomic deposited piezometers. The first of these techniques has been employed in aerospace engineering (Liu et al. 1997). This technique was developed to measure pressure applied on a surface covered by the paint. The spatial resolution of the technique is affected by the coverage of the surface by PSP and the resolution of the detection system (McLachlan and Bell 1995). It is based on the emitted light wavelength from a paint containing luminophore. For example, in the case of air pressure measurement, emitted wavelength is affected by the increase in the number of oxygen molecules colliding on the paint surface, when more pressure is applied. This technique has a number of shortcomings for micro-model applications. The pressures estimated at the pore scale are in the range of a few hundreds to a few thousands pascal. But pressure-sensitive paints are usually used to measure pressures in the range of hundreds of kPa–MPa. Recent work on pressure-sensitive paints has led to the development of paints based on Diketopyrrolopyrrole (DPP8), which are sensitive in the range of 700–5000 Pa (Chung et al. 2015). We performed tests on the use of this wax-type paint. We found that it cannot be used in micro-model applications. PDMS tends to swell when in contact with this organic chemical. In addition to that, DPP8 didn’t seem to react in the pressure range noted by (Chung et al. 2015). The second technology that could allow the pore-scale measurement of a fluid pressure is based on the piezoelectric properties of certain materials. Atomic deposition of piezoelectric sensors (Kuoni et al. 2003) on one of the micro-model inner surfaces would ideally give us a complete mapping of the pressure field inside the micro-model. However, manufacturing of such models is challenging and extremely expensive. This is probably the reason that such models have not been developed for pressure measurement in micro-models until now. Therefore, we developed a cost-effective pressure measuring technique that would allow us measuring pressure at the pore scale. The purpose of developing such a micro-model is to obtain pore-scale pressure values that can be used to increase our understanding of two-phase flow and, in particular, the formation and remobilization of discontinuous non-wetting phase.

In this paper, we describe the fabrication of a micro-model, made of PDMS, with integrated fibre optic piezometers. We demonstrate that these sensors provide pore-scale pressure measurements during two-phase flow. We show that the sensor shows an almost linear pressure distribution during steady-state single-phase flow. The variation of pore pressure with time at each sensor location clearly shows the effect of minor and major pore-filling events as well as breakthrough of the fluids when they reach the micro-model outlet.

## Fabrication Procedure

### Micro-model Design and Geometric Characteristics

**Fig. 1:**
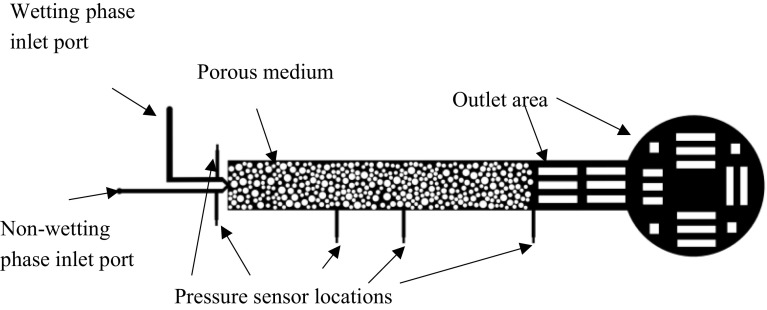
Micro-model image. The black colour corresponds to pore network of the micro-model. The pressure sensors locations are shown by five small bars. During two-phase flow experiments, non-wetting phase is injected from the lower inlet and the wetting phase is injected by the upper one

The micro-model used in this study had three parts. The main part was the pore network formed by the void space between two slabs of PDMS connected by a large number of randomly distributed non-overlapping cylindrical pillars (shown as white circles in Fig. 1), having similar pore topology as in previous works (Ng et al. 1978; Reddi 1998; Tallakstad et al. 2009). The diameters of the cylinders, which represent the solid phase of the porous medium, were chosen from a random distribution, with maximum and minimum values of 80 and 20 $\upmu \hbox {m}$, respectively. The cylinders all had the same height equal to the desired depth of the micro-model network. In order to accommodate the fibre optic piezometers (described in detail in Sect. 2.3), we needed a minimum depth of 320 $\upmu \hbox {m}$. The network’s dimensions were 4 mm by 26 mm, and it had a porosity of 50%.

The second part of the micro-model was the inlet and outlet ports. This model had two separate inlet ports, one for each phase and one common outlet port. The two inlet ports allow for the simultaneous injection of two fluid phases from the same side, for the investigation of ganglia behaviour, similar to the works of (Avraam and Payatakes 1995, 1999). The outlet port is in the middle of the outlet area; it is not shown in Fig. 1 as it is on the other PDMS slab.

The third part of the micro-model was a set of measurement ports where the fibre optic piezometers can be inserted. In this design, we had five ports, two for each fluid at the inlet, one at the outlet and two along the micro-model. In Fig. 1, the positions of the sensors are shown as black vertical lines.

**Fig. 2:**
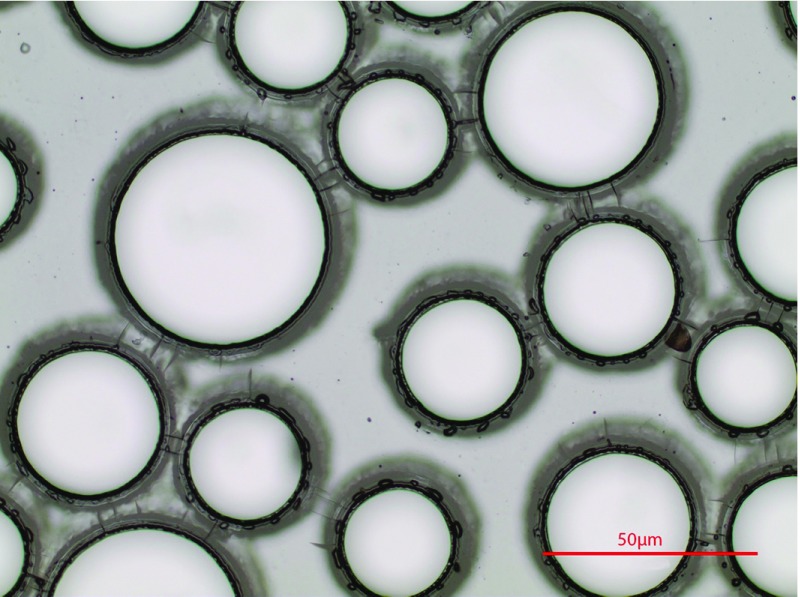
Microscope image of the silicon wafer after development

### Soft Lithography

Manufacturing of PDMS micro-models was based on soft lithography (Quake and Scherer 2000; Markov et al. 2010). Soft lithography is a technique that is based on the use of elastomeric materials to produce micro- and nanoscale structures. A detailed description of the manufacturing procedure can be found in Karadimitriou et al. (2013). Here a short summary is given.

#### Mould Manufacturing

First, a silicon wafer was spin-coated with the liquid polymer SU8-2025 (MicroChem) according to the recipe, proposed by the supplier. The thickness of the coating was dictated by the target depth of the micro-model, i.e. 320 $\upmu \hbox {m}$. This could not be applied in one step and had to be made in four layers. Given the viscosity of SU8-2025, each layer thickness was limited to 80 $\upmu \hbox {m}$. First, a layer of 80 $\upmu \hbox {m}$ was spin-coated and the wafer was soft baked at 65 and 95 $^{\circ }$C, for 3 and 8 min respectively. This was repeated three more times to achieve the required thickness. In order to ensure the adequate evaporation of the polymer’s solvent, after the first layer, the soft baking time was increased by 20% for every baking cycle.

The digitally generated pore network, described in Sect. 2.1 and shown in Fig. 1, was printed on a transparency**.** The transparent mask was placed on the soft baked photoresist coating and was exposed to UV light. The exposure times were calculated based on the recipe of SU-8 and the energy of the UV lamp for a film of 320 $\upmu \hbox {m}$. This was followed by the post-exposure baking (hard baking) at 65 and 95 $^{\circ }$C, for 4 and 15 min respectively.

After exposure, the silicon wafer was developed for 30 mins with SU-8 developer (MicroChem). During this step, the unexposed photoresist was dissolved by the developer. An image of the result is shown in Fig. 2. To check whether the thickness was correct, the wafer was systematically measured with a profilometer. Afterwards, the wafer was baked at 150 $^{\circ }$C for 30 min. Finally, the wafer was silanized in a vacuum chamber with trichloro-(1H,1H,2H,2H-perfluorooctyl) silane. The final product was a mould bearing the negative of the image shown in Fig. 1.

**Fig. 3:**
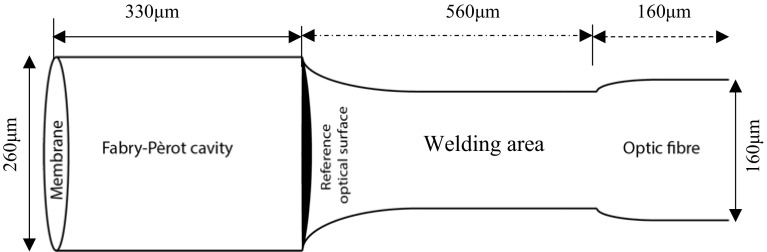
Schematic representation of a fibre optic pressure sensor. The pressure sensor and 300 $\upmu \hbox {m}$ of optical fibre were embedded into the micro-model wall

#### Micro-model Production

The wafer was used as a mould for the fabrication of the micro-models. A mixture of 50 g of PDMS and 5 g of curing agent was prepared. The mixture was degassed in a vacuum chamber, before it was poured carefully into a petri dish containing the mould. In another petri dish, we poured enough mixture to make a solid slab for covering and sealing the micro-model. The two petri dishes were degassed in a vacuum chamber before they were cured in the oven at 68 $^{\circ }$C. The two cured parts were removed from the petri dishes and placed in a small chamber for the silanization step (Zhou et al. 2010). Usually, the silanization step is done after the model is sealed (Karadimitriou et al. 2013). However, in order to prevent the potential effect of this treatment on the pressure sensors, which could affect their performance, we performed the silanization process prior to the insertion of the pressure sensors and the sealing of the micro-model.

The final steps in the manufacturing of the micro-model were the insertion of the pressure sensors and the bonding of the two slabs of the micro-model. First, the sensors were placed by hand in the pre-set locations. Finally, the two slabs were bonded together with a corona discharge (Haubert et al. 2006) and then the micro-model was kept in room temperature for 24 h for the bonding to mature. Although the silanization step preceded the bonding of the micro-model, we didn’t encounter issues with sealing the micro-model.

### Fibre Optic Pressure Sensors

Obviously, a pressure sensor for pore-scale measurements must have the same or smaller dimensions as the pores. Traditional pressure sensors have at least one order of magnitude larger dimensions, usually in the range of millimetres. Therefore, they cannot fit in the pore space of a micro-model. Fibre optic pressure sensors (Bao et al. 2013) can be used for measuring pressure at this scale. These are miniature fibre optic piezometers (FOP - MIV) (Smartec), which are used for fluid pressure measurements in live tissues. The sensors used in this micro-model are the FOP-M260-SHEATHED model (Fig. 3). These sensors have a diameter of 260 $\upmu \hbox {m}$, and they are covered with a protective sleeve, which gives an overall diameter of 320 $\upmu \hbox {m}$.

A schematic presentation of the pressure sensor is shown in Fig. 3. The sensor consists of the following parts: a cylindrical cavity formed between a flexible membrane, with a reflective inner surface, and a reference optical surface at the optical fibre end. This configuration constitutes a Fabry–Pèrot interferometer (Totsu et al. 2005). The fluid pressure acting on the membrane deforms it. This consequently changes the cavity’s length. The light, emitted from a light source travels through the optical fibre into the cavity, reflects the membrane’s surface and finally travels back though the optical fibre to the spectrometer. The measured spectrum of the light, altered by the change in cavity length, is translated into pressure.

**Fig. 4:**
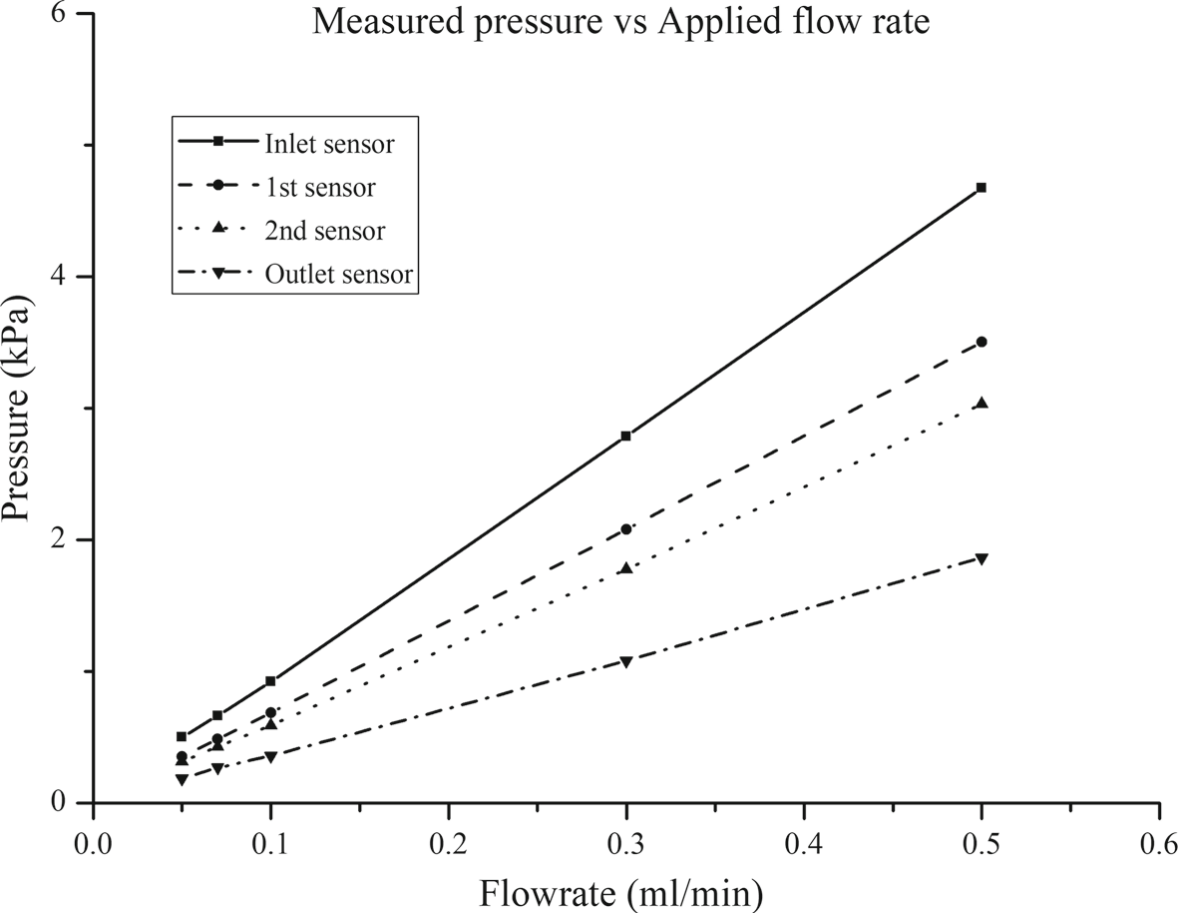
Pressure measured by each sensor at various flow rates

Their measurement range is from − 40 up to 40 kPa, with a resolution of 40 Pa and accuracy of 0.6% of the full range, which makes them ideal for monitoring pressure at the pore scale. Moreover, the acquisition rate of 250 Hz is sufficient for fast monitoring of pressure changes commonly encountered in dynamic two-phase flow experiments. The sensors were tested in various ways. First, we used them to measure air pressure under controlled conditions. In another test, we placed a sensor at the bottom of a graded cylinder filled with one metre of water. The level of water was reduced by increments of 5 cm and the sensor readings were collected. We found a strictly linear variation of the sensor readings with the height of the water. Next, once the sensors were embedded in the micro-model, we saturated the micro-model with the wetting phase and subjected it to a known pressure. We then recorded sensors readings under no flow conditions. In all cases, deviations of the pressure measurements from known values were in the range of the resolution (i.e. 40 Pa). Finally, we checked pressure measurements during steady-state single-phase flow experiments at known flow rates. Experiments are described shortly. Results showed that the sensors response with flow rate was linear, as it is shown in Fig. 4.

## Flow Experiments

### Experimental Set-up

**Fig. 5:**
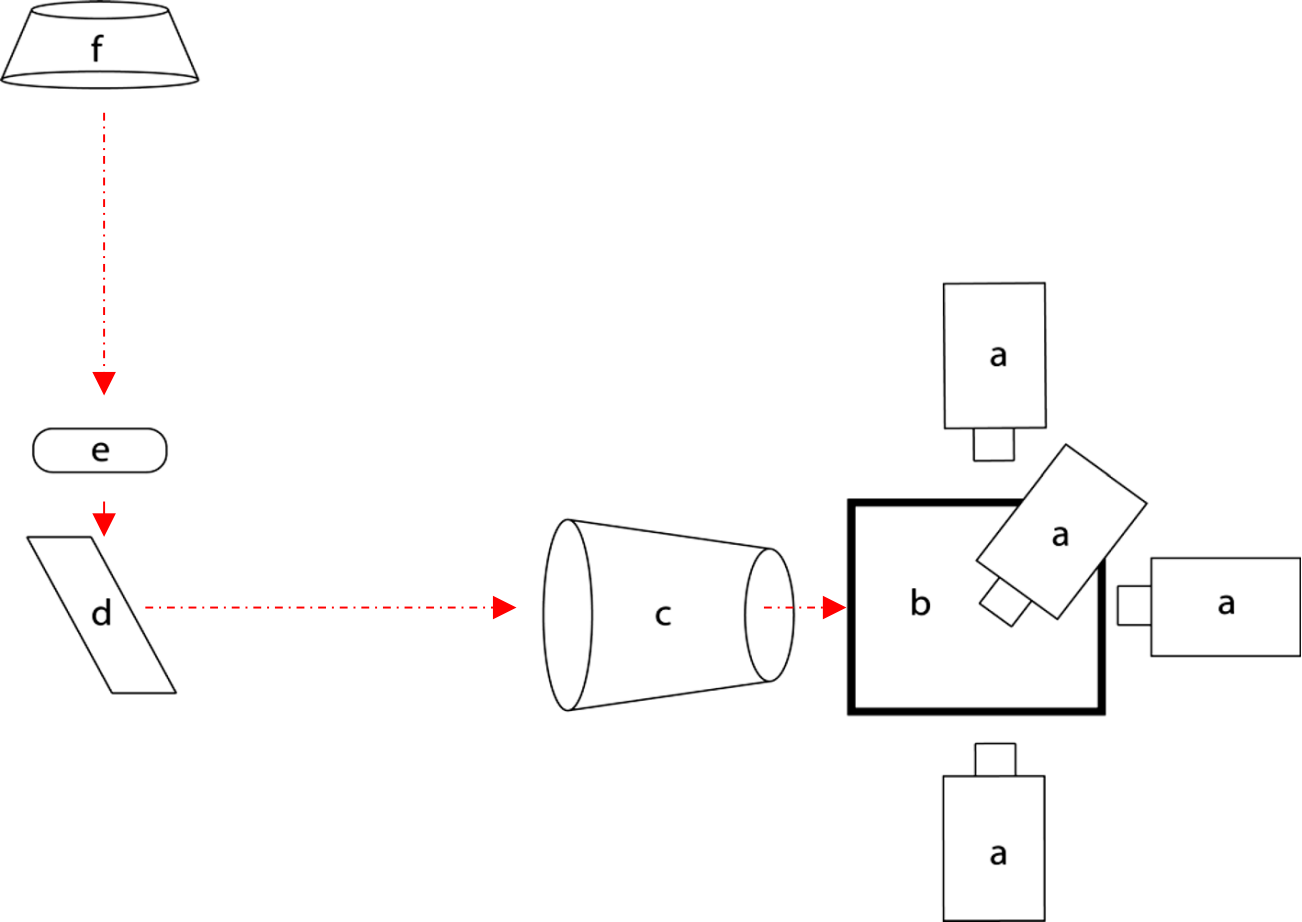
The experimental set-up: (*a*) high-resolution CMOS camera, (*b*) box with three beam splitters, (*c*) optical lens, (*d*) prism, (*e*) micro-model location, (*f*) light source

The experimental set-up is shown in Fig. 5. It consisted of (a) four CMOS cameras, (b) a box with three beam splitters, (c) an optical lens, (d) a prism and (f) a light source.

The set-up can be considered as an “open air microscope”, which allows the user to observe in real time the whole micro-model at high spatial and temporal resolutions. The micro-model is illuminated with a collimated light source, and the transmitted light is diverted thought a prism to an optical lens and the beam-splitter box. This box with three beam splitters produces four identical images of the micro-model going out in four perpendicular directions. Each of those images is projected onto the CMOS of one optical camera placed around the box. The visualization parts are described in detail in previous works (Karadimitriou et al. 2014a, b).

**Fig. 6:**
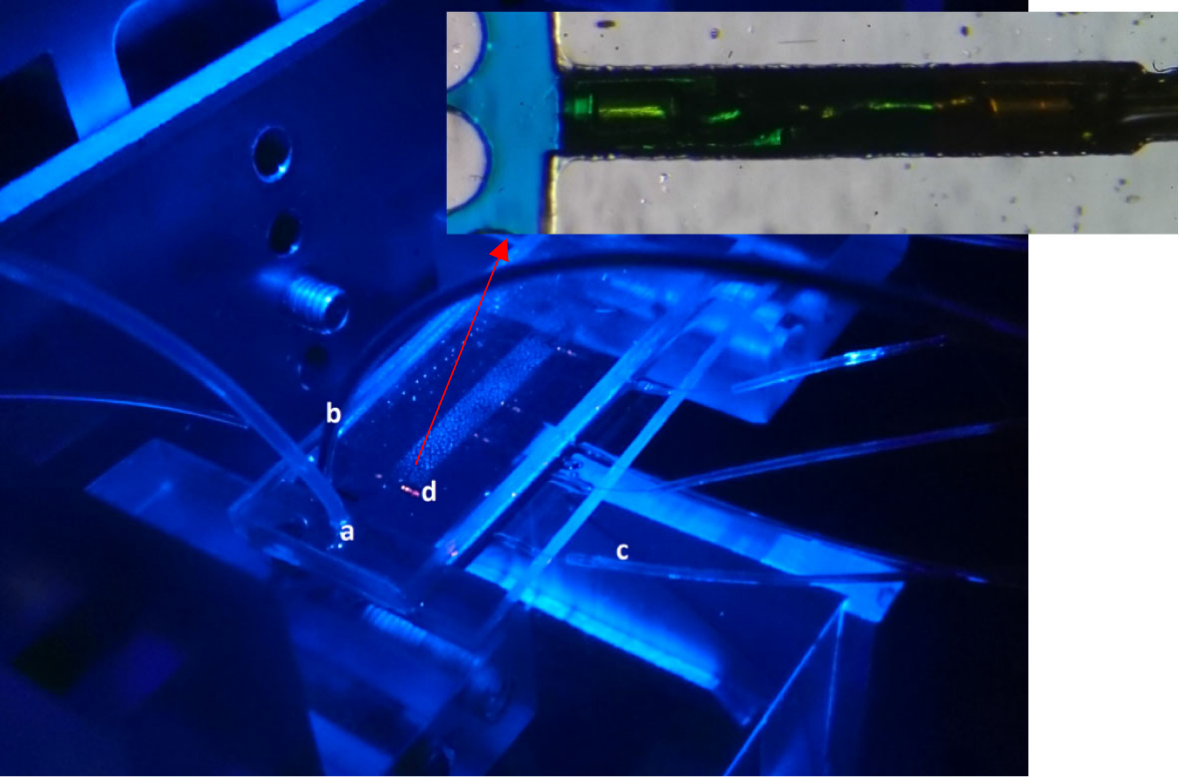
Picture of the micro-model on the experimental set-up: (*a*) wetting phase injection tube, (*b*) non-wetting phase injection tube, (*c*) fibre optics, (*d*) pressure sensor. The inset shows the sensor embedded in the micro-model wall, with its tip in contact with the fluid in the pore

Pressure measurement was performed by means of five fibre optic piezometers, integrated into the micro-model (Fig. 6). These sensors are connected to a signal conditioner, which is equipped with FPI-HR Module. This module is using a polynomial fit instead of a traditional regression fit to ensure better accuracy. The locations of pressure sensors are shown in Fig. 1.

The final part of the experimental set-up is the flow control unit. The injection of fluids is controlled by two syringe pumps (Harvard Apparatus Pump 11 Pico Plus Elite). To ensure the accuracy (± 0.35%) of the injection rate and the volume of the injected phases, the syringes used were air-tight 5-ml Hamilton glass syringes. The syringes were connected to the micro-model with 1/16” FEP tubing (Inacom).

### Experimental Procedure

Polydimethylsiloxane (PDMS) is naturally hydrophobic. Therefore, Fluorinert FC-43 was used as the wetting phase and water dyed with Ecoline 237 as the non-wetting phase. Properties of the two fluids are found in Table 1.

**Table 1:** Fluid properties used in the two-phase flow experiments (at 21 $^{\circ }$C)

Fluid	Viscosity $(\hbox {Pa}\cdot \hbox {s})$	Density $(\hbox {kg/m}^{3})$	Interfacial tension (mN/m)
Water	$0.9\times 10^{-3}$	1000	58
Fluorinert	$4.7\times 10^{-}$3	1860	

First, we performed single-phase flow experiments, during which the model was filled with Fluorinert FC-43 (the wetting phase) at all times. In these experiments, we injected Fluorinert at five different flow rates with pore-scale Reynolds number ranging from 1.3 to 13. The aim of these experiments was to test the response of the pressure sensors under flow conditions. Moreover, these data were used for calculating the permeability of the micro-model.

In our two-phase flow experiments, the micro-model was initially saturated with the wetting phase (Fluorinert FC-43). Then we performed a primary drainage by injecting dyed water (non-wetting phase), from one of the inlet ports, at a flow rate of 0.1 ml/min until breakthrough occurred. After breakthrough the imbibition was initiated by injecting Fluorinert at the same flow rate (0.1 ml/min) from the same side of micro-model. Both water and Fluorinert could freely leave the micro-model from the outlet area.

## Results

### Single-Phase Flow Experiments

**Fig. 7:**
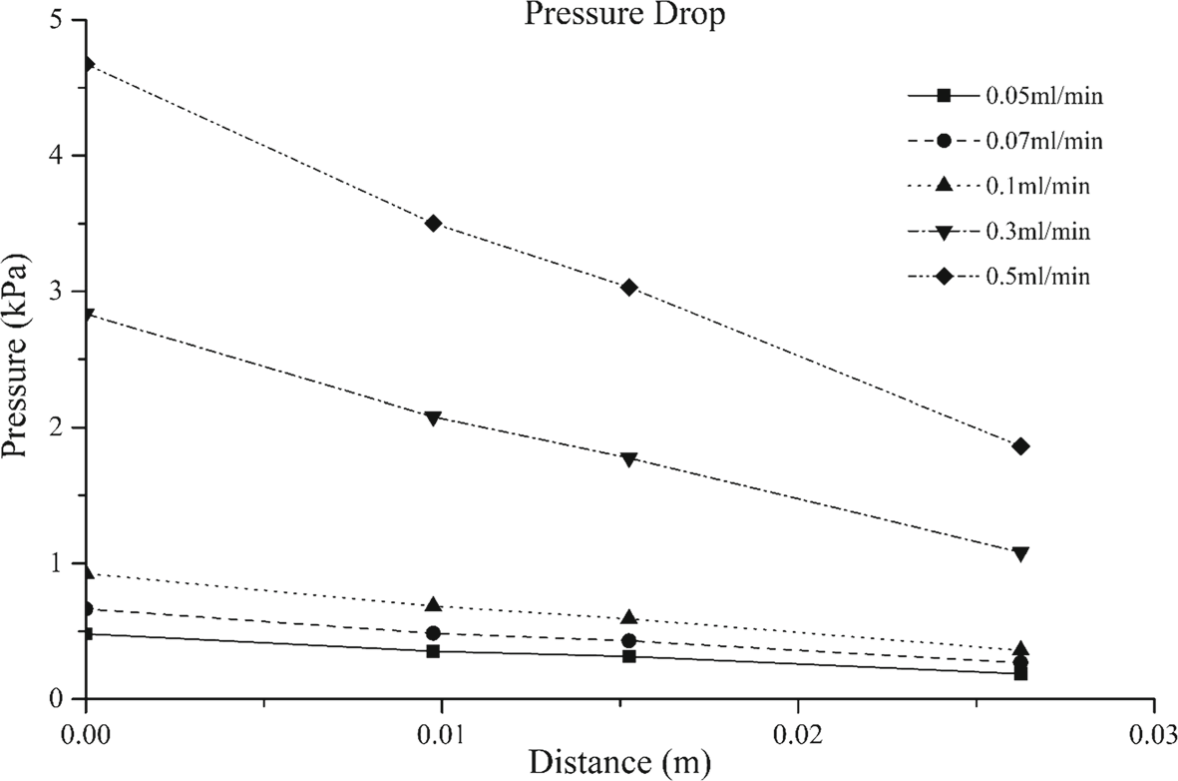
Pressure drop over the micro-model during single-phase flow. During all five different flow rates, the pressure drop remained linear

The single-phase flow experiments were performed at five different flow rates, mentioned above. Figure 7 shows the pressure variation along the micro-model. Note that the value of only one of the two inlet sensors is shown as they measured the same pressure value. In calculating the distance between the inlet sensor and the first sensor in the micro-model, we have taken an approximate tortuous path within the pores. The distance between all other sensors was measured as the straight line. It is evident that the pressure varied almost linearly for all flow rates, as expected. This result proves that the sensors measured the correct pressure during flow conditions. Finally, knowing the pressure drop across the micro-model for each flow rate, the permeability was calculated using Darcy’s law and was found to be $2.7 \pm 0.04\times 10^{-10}~\hbox {m}^{2}$.

### Two-Phase Flow Experiments

#### Drainage

**Fig. 8:**
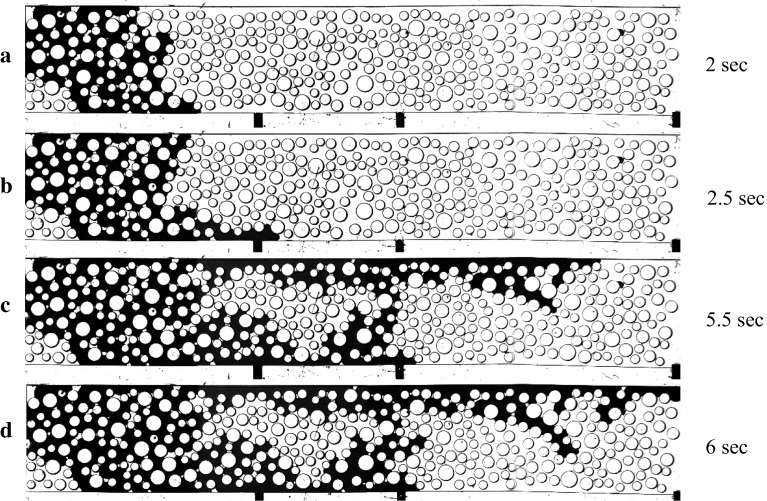
Images acquired during the primary drainage. The wetting phase, with which the micro-model was initially saturated, is shown in white, and the non-wetting phase is shown in black colour

**Fig. 9:**
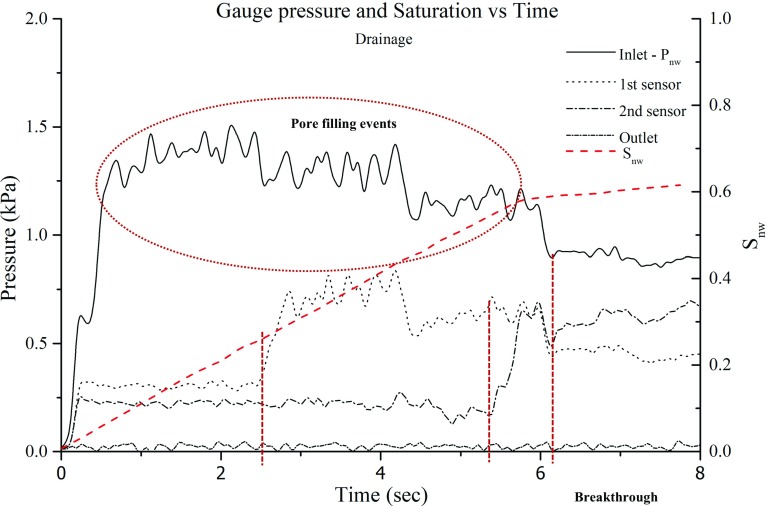
Evolution of pressure and non-wetting phase saturation over time at the four measurement locations during drainage

Figure 8 shows images of the micro-model at four different times during drainage. The flow rate of the non-wetting phase (shown in dark colour) was kept constant at 0.1 ml/min $(\hbox {Ca}= 3.7\times 10^{-5})$. One of the inlet ports was closed. At the initial stages, the invasion formed a front but later evolved into two fingers on the two sides of the micro-model. Measured pressure values of four sensors, as well as the water saturation averaged over the whole micro-model $(\hbox {S}_{\mathrm{nw}})$, are plotted against time and shown in Fig. 9. The outlet pressure remained constant until the breakthrough of the non-wetting phase occurred. Comparing the measured pressure curves with the increase of the non-wetting phase saturation, it is evident that the sensors responded to the spread of the non-wetting phase in the micro-model. We note that the overall pressure drop (i.e. the difference between the inlet and outlet pressures) decreased as more and more water displaced Fluorinert. This is due to the fact that the water viscosity is lower.

When the non-wetting phase reached the first sensor inside the micro-model at 2.4 s (indicated by the first red dotted line in Fig. 9), the pressure increased to around 800 Pa, due to the fact that the non-wetting phase pressure is larger than the wetting phase pressure, because of capillarity. The pressure at this location remained elevated throughout the duration of drainage, but gradually decreased over time, again because water has a lower viscosity. A similar rise occurred in the pressure measured by the second sensor, when the non-wetting phase reached it at 5.4 s (indicated by a second dotted line). Further, we notice a step drop in pressure readings at the inlet and at sensor 1 around 4.2 s when the two fingers shown in Fig. 8c were formed. Finally, the pressure at inlet and the two sensors inside micro-model dropped at 6.2 s (indicated by the third red dotted line), when the upper non-wetting phase finger (Fig. 8d) reached the outlet. Note that after breakthrough of the non-wetting phase, its saturation remained almost constant. We also notice an unexpected decrease in the pressure at the first sensor such that it becomes smaller than the pressure at the second sensor. We cannot explain this reversal of pressure gradient based on images, as the pore occupancy does not change.

**Fig. 10:**
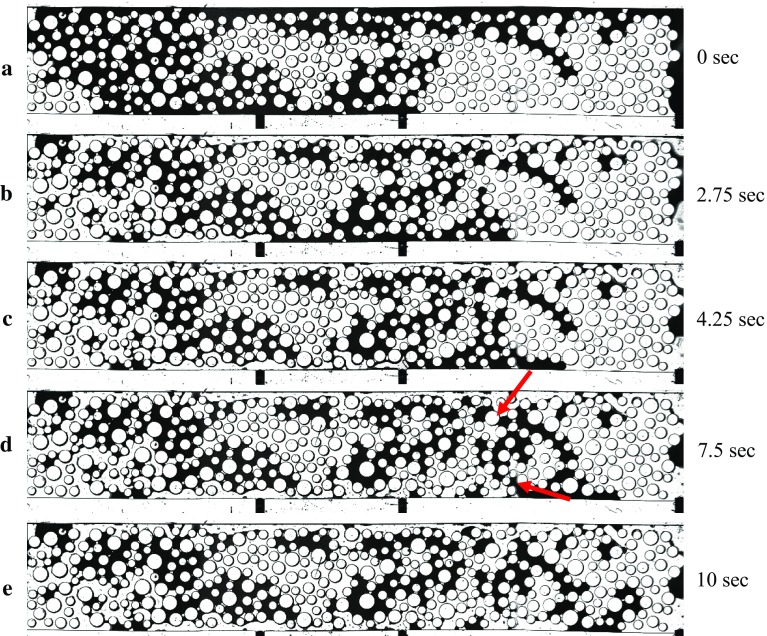
Images acquired during the main imbibition. The wetting phase, shown in white, displaced the non-wetting phase, shown in black, from left to right. The red arrows mark the throats which control the wetting phase flow, which caused an increase in pressure between 7.6 and 8.2 s

In Figure 9, we notice some fluctuations in the measured pressures. This behaviour is due to various pore-filling events, such as Haine’s jumps and local imbibition during drainage. Note that pressure acquisition rate was much higher than the imagining frame rate used in these experiments. So, although we could measure pressure fluctuations, we could not capture Haine’s jump.

#### Imbibition

**Fig. 11:**
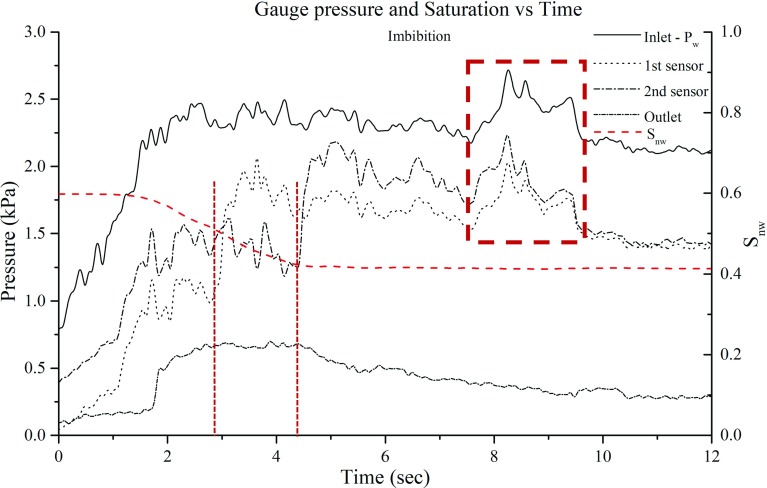
Evolution of pressure and non-wetting phase saturation over time at the four measurement locations during imbibition. The red rectangular indicates the peak in pressure caused as the wetting phase invades two bottleneck pores shown in Fig. 10d

After breakthrough occurred, we ended the injection of non-wetting phase (water) and flow stopped. As a result, the non-wetting phase pressure at the inlet dropped to 760 Pa, as it can be seen at the start of imbibition measurements in Fig. 11. Then, imbibition was initiated by injecting Fluorinert (wetting phase) from one inlet port at a flow rate of 0.1 ml/min (Ca = $1.69 \times 10^{-4}$); so, again the flow was from left to right. The distribution of fluid phases, at key moments of imbibition, is shown in Fig. 10. The variation of pressure measured by four sensors and the change of non-wetting phase saturation are shown in Fig. 11. Similar to drainage, the pressure increased at the beginning of the cycle to provide the pressure gradient needed for the flow of the two liquids. Initially, the pressure drop was small as the non-wetting phase (water) formed a continuous phase and could be displaced by Fluorinert. However, after 4.4 s, the non-wetting phase became discontinuous and could not be removed anymore. The wetting phase saturation was still less than 50% and it remained below 60%. So, the overall relative permeability was low and a relatively high pressure drop was needed for maintaining the flow of the wetting phase. The wetting phase reached the first pressure sensor at 2.75 s after the imbibition started and the second sensor at 4.25 s (Fig. 10b, c respectively); at those times the corresponding pressure showed a step increase.

As in the case of drainage, the pore-filling events caused fluctuations in the measured pressures. An important event is the occurrence of a pressure peak, recorded by at the inlet sensor and the two inside sensors at 7.6 s, and a subsequent decrease at 8.2 (see the period shown by a red rectangle in Fig. 11). This pressure increase was due to the fact the discontinuous non-wetting phase had blocked the width of the micro-model in the areas shown by red arrows in Fig. 10c. The wetting phase pressure had to build up for it to be able to invade the two smallest pores marked by red arrows. Once those pores were opened (see the next image, Fig. 10d), the pressure dropped as a large number of pores were contributing to the flow of wetting phase.

## Conclusions

Direct measurement of pressure at the pore scale has been a challenge in experimental studies of two-phase flow. In this work, we have succeeded in incorporating a micro-pressure transducer in a micro-model allowing us to measure pore pressure within the micro-model.

Our experimental results show that these types of micro-models can contribute to a better understanding of two-phase flow. Detailed pressure data reveal small-scale events that occur during drainage and imbibition. Formation of fingers, overcoming a bottle neck formed by pores that have to be invaded during imbibition or drainage, and breakthrough of a phase lead to clear rise or drop in measured pore pressures. Trapping and mobilization of non-wetting phase, in the form of ganglia and/or blobs, are controlled by these pore-scale events. Therefore, these types of micro-models can lead to a better understanding of this phenomenon.

Finally, obtaining pressure information at this scale will also contribute to the testing of pore-scale numerical models. Previous works (Kunz et al. 2015) show a time scale mismatch between experimental and numerical works. Pore-scale pressure measurements can be used to calibrate and correct these models, leading to more accurate numerical results.
